# Mass Psychogenic Illness: Demography and Symptom Profile of an Episode

**DOI:** 10.1155/2016/2810143

**Published:** 2016-05-16

**Authors:** Binoy Krishna Tarafder, Mohammad Ashik Imran Khan, Md. Tanvir Islam, Sheikh Abdullah Al Mahmud, Md. Humayun Kabir Sarker, Imtiaz Faruq, Md. Titu Miah, S. M. Yasir Arafat

**Affiliations:** ^1^Department of Medicine, Faridpur Medical College, Faridpur, Bangladesh; ^2^Pulmonology, Bangabandhu Sheikh Mujib Medical University, Bangladesh; ^3^Internal Medicine, Bangabandhu Sheikh Mujib Medical University, Bangladesh; ^4^Pediatrics, Sheikh Sayera Khatun Medical College, Gopalganj, Bangladesh; ^5^Sheikh Sayera Khatun Medical College, Gopalgang, Bangladesh; ^6^Medicine, Dhaka Medical College Hospital, Dhaka, Bangladesh; ^7^Department of Psychiatry, Bangabandhu Sheikh Mujib Medical University, Bangladesh

## Abstract

*Background*. Mass psychogenic illness has been a recurrent phenomenon in Bangladesh over recent times.* Objectives*. This study was aimed at investigating the demographic characteristics and symptom profile of an outbreak of mass psychogenic illness occurring in a girls' high school.* Methods and Materials*. In 14 April 2013, a total of 93 students of a girls' high school suddenly developed various symptoms following intake of tiffin cake which resulted in panic and hospital admission. A descriptive, cross-sectional observational survey was done to define various characteristics of the outbreak.* Results*. No organic explanation for the reported illnesses was found. 93 female students were included who were hospitalized during the incident. Trigger factor was found in 98% of students. Most of the students were 13 years old. Average interval between exposure to the trigger and onset of symptoms was 151.5 minutes. Commonest symptoms were abdominal pain (83%), headache (73%), chest pain (69%), body ache (63%), nausea (69%), and generalized weakness and fatigue (61%). Hospital stay following the incident was about 12 hours on average.* Conclusion*. To avoid unnecessary panic in the community a prompt, coordinated response is important in resolving widespread community anxiety surrounding these episodes.

## 1. Introduction

Mass psychogenic illness (MPI) is a not a very rare phenomenon anymore. It is probably more common than the reported incidences and imposes a significant financial burden and unexpected management difficulties for the emergency department [1]. Affected schools or occupational sites are often closed for days or weeks [2]. It can be defined as “the rapid spread of illness signs and symptoms affecting members of a cohesive group, originating from a nervous system disturbance involving excitation, loss, or alteration of function, whereby physical complaints that are exhibited unconsciously have no corresponding organic etiology” [3]. Outbreak of mass psychogenic illness often starts with an environmental trigger such as a bad smell, a sound, a suspicious looking substance, or something else that makes people in a group believe that they have been exposed to a danger and they start to experience symptoms of illness [4, 5]. An index case sometimes has organic problem. It has unique characteristics—symptoms without any plausible organic cause, transient and benign, occurring in a segregated group, presence of extraordinary anxiety, spread via sight, sound, or oral communication; the spread moves down the age scale, more affecting the females [6]. Common symptoms are nausea, dizziness, fainting, headache, abdominal pain, hyperventilation, cough, fatigue, drowsiness, weakness, watery eyes, chest pain, vomiting, and so forth [6–9]. Investigators failed to conclude regarding any predisposing factor of an episode, but psychological factors, environmental factors, different stressors, conflicts, lower education, lower socioeconomic status, minority race, and history of abuse or trauma may be involved [10–19]. Responders of emergency management and the initial media reports of unexplained illness are believed to amplify the phenomenon by spreading threatening information and lead more people to report symptoms [20]. Brief duration of illness, sudden onset without specific time, place, or person, and lack of opportunity for controlled experiment have made it a difficult field for the researchers. This study was aimed at reporting the demographic characteristics and symptom profile of an outbreak of mass psychogenic illness occurring in a girls' high school at Gopalganj in Bangladesh in April 2013.

## 2. The Episode

The incidence occurred in a girls' high school in the morning of 14 April 2013 after serving tiffin to the students like every other day. Few students of class five started feeling unwell within few minutes of taking the tiffin while playing in the playground. They were brought to the headmaster's room immediately. As the news spread among other students, some of the other students also started to feel sick. They were brought to the emergency department of nearby hospital. Subsequently some of the students who carried the sick students to hospital also felt unwell. Most of the students complained of bad odor or bad taste of the cake. All affected students were admitted. Treatment was given after a triage done by the hospital consultants. Counseling was done repeatedly to the sick students. Assurance and reassurance were also given to the parents and relatives. Within a short period of time media men and more anxious relatives rushed to the hospital. The situation was getting worse and local administration along with police department got involved to control the situation. Political leaders, social workers, and many curious people also came. Reports of a mysterious illness were being broadcasted in all the major television channels. Rumor of death of two to three students further worsened the situation. Situation was gradually controlled with the combined efforts of the doctors, nurses, other hospital staff, administrative personnel, and police. Some students feeling well left the hospital. Before evening, many students recovered and were discharged. Some students needed to stay in hospital up to the next day and few up to the following day. Some students became ill again after returning home and needed readmission. Three students were referred to the nearest tertiary care hospital because of deep concern of the relatives whose anxiety could not be alleviated by existing facilities. The supplied cake was considered as the environmental trigger of the episode and sample of cake was sent for microbiological and toxicological examination.

## 3. Materials and Methods

This cross-sectional observational survey was done in 250 Bedded General Hospital, Gopalganj, from 14 to 20 April 2013 to describe the characteristics of the outbreak, timeline of events, symptoms experienced, risk factors, and community perception of the event. An ethical clearance was taken from the concerned authority for the purpose of the study considering an emergency.

Diagnosis was confirmed by an internist, pediatrician. In some cases, relevant investigations were done to exclude organic causes. A detailed questionnaire was prepared promptly after the episode. All the students who were admitted with the illness during this period were included in the study. Those who came to hospital out of fear of illness and having no symptoms were excluded. Information was gathered from the sick students on their recovery and from their attending guardians by face to face interview. Help from the teachers, guardians, and administrative and health personnel was received during data collection and managing the hospital rush during the episode. Data were managed and analyzed by Statistical Package for Social Science (SPSS, V 16) version 16 and Microsoft Excel 2007 version.

## 4. Results

In the study, total 93 students were included who were hospitalized following the incident. Age distribution of the students showed that most students (34%) were 13 years old. Next commonest age group was 14 years (28%) and 12 years (15%), respectively (Table 1).

Average interval between exposure to the trigger and onset of symptoms was 151.5 minutes. About 27% of students developed symptoms within 45–60 minutes following exposure and 26% developed symptoms after 60–90 minutes. Some students (15%) developed symptoms very rapidly (within 0–15 minutes).

The trigger factor of the outbreak was consumption of the cake which was found in 98% of the ill students. An abnormal smell or taste or both were noticed in the supplied cake by 88% of students, whereas 5.4% of students felt ill spontaneously while playing in the ground. 20% of the students felt ill by only seeing other ill students in the school. During transfer of the sick students, 63% of students who volunteered and came in direct contact with the ill students felt sick. 11% of students felt sick while watching television telecast of the incident. All students reported exam ahead within one month (Table 2).

Affected students had various symptoms. Commonest symptoms were abdominal pain (83%), headache (73%), body ache (63%), nausea (69%), generalized weakness and fatigue (61%), and chest pain (69%). Among other complaints, 58% felt burning sensation of body, 45% complained of dizziness, and 31% had dry mouth (Table 3).

**Figure 1:**
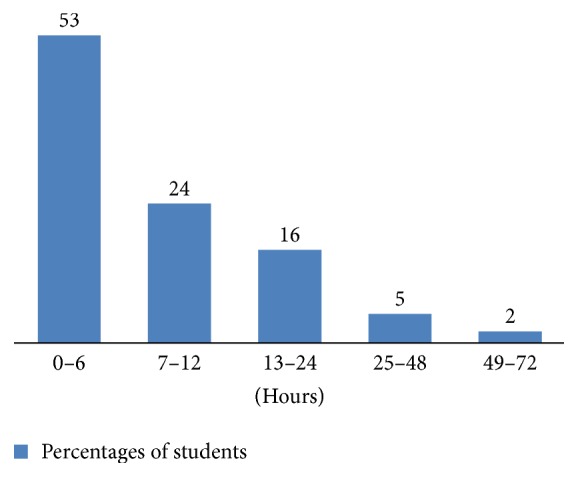
Duration of hospital stay of the respondents of the episode.

Around 53% of the students left the hospital within 6 hours of admission. About 24% left the hospital between 7 and 12 hours, 16% between 13 and 24 hours, and 5% between 25 and 48 hours of admission. Only 2% required hospital stay up to 49–72 hours (Figure 1). Hospital stay following the incident was 12.3 hours on average. A relapse of the attack needing readmission was present in 18% of students.

## 5. Discussion

Every year, an estimated four to six separate outbreaks of mass psychogenic illness reach public attention, but the actual frequency is not known [21]. Our reported episode gives a classical example of mass psychogenic illness with an identifiable triggering factor, rapid onset of illness, rapid recovery, and absence of any organic illness. Age distribution of the students shows that most students were 13 years old. This distribution is consistent with the characteristics of mass psychogenic illness and also the current published reports [6]. Various studies published between 1973 and 1993 show that schools are very common place for mass psychogenic illness accounting for more than 50% of the events [22].

Among the admitted 93 students, 98% took the cake which was supplied and 88% noticed either bad taste or bad smell or both. This had acted as an environmental trigger factor to initiate the episode. In many reported episodes the trigger factors are found and were like tiffin biscuits, following vaccination, bad environmental smell, and so forth [2, 23, 24]. A clear index case is often the source of symptoms from whom the symptoms spread to others [25, 26]. The index case may or may not be suffering from organic illness. In this incidence we could not clearly identify the index case, but five girls initially became ill spontaneously and they were not suffering from any previous organic diseases. The result of sample of cake sent for microbiological examination did not prove any association with toxic or infectious agent. Also, teachers and other students who took the same cake at the same time did not experience any abnormal symptom. Preliminary investigations did not indicate any organic illness in our subjects.

Onset of symptoms following exposure to environmental trigger is rapid in mass psychogenic illness. In our study, average time of onset following trigger exposure was 151.5 minutes. The onset was very rapid in some students. Most of the students started to show symptoms within the period of 45–90 minutes after the trigger. This may be due to the fact that they were helping during transportation of fellow ill students to the hospital and coming in direct contact and got ill. Contagion is increased by being close to affected and unaffected persons, reassembly of the group such as being put in the same ward in a hospital, and “line of sight” transmission [2, 27]. Some students were found to be affected very lately. Another factor might have been the intensive media attention which heightened the collective anxiety and contributed to the second cluster of cases.

A very rapid spread of symptoms which frequently includes hyperventilation or syncope with minimal physical findings often occurs in groups under physical or psychological stress. Unnecessary dramatic and prolonged media coverage on the issue frequently enhances such outbreaks [28–33]. In our outbreak, exam schedule was ahead within one month for all students which might have acted as stressor and significant number (84%) of ill students came to school without taking breakfast which might have acted to precipitate the outbreak. Commonest symptoms in our study were abdominal pain (83%), headache (73%), body ache (63%), nausea (69%), generalized weakness and fatigue (61%), and chest pain (69%). Among other complaints, 58% felt burning body, 45% complained of dizziness, 31% had dry mouth which is consistent with other studies [8, 9, 18, 24, 25].

Around 53% of the students left the hospital within six hours of admission. Recovery is rapid in MPI. In our study, average hospital stay was 12.3 hours, but some students (2%) had to remain in hospital even for 3 days. Recurrent attack has been described in some studies [23, 25]. In our study, 18% students experienced recurrence of symptoms and needed readmission. Once the diagnosis is determined, reassuring patients is the primary therapy. Separating them can be beneficial. Most patients experience rapid resolution of symptoms once they are removed from the environment in which the outbreak started [6].

There may exist a relation of MPI episode and exposer of low level environmental toxins as reported in studies [34], but in this reported episode there was no association to be noted and mentioned as it was duly searched for the possible and assessable causes based on clinical, laboratory investigations and microbiological examination and all outcomes revealed nothing contributory regarding the episode.

A teamwork of internists, pediatricians, hospital administrators, and nurses was key to the successful handling of the situation. The idea that the outbreak was psychogenic is very clear but because of intense anxiety in the parents, investigations were undertaken to rule out any organic cause. With any approach to mass psychogenic illness, the goal should be to restore the community to normal functioning as quickly as possible. In our study we found that most of the guardians (82.8%) thought the outbreak was due to unknown poisoning from the cake. The rest of them thought it was some other illness. None believed it as any supernatural act. In a study done in various districts of Bangladesh in 2007, 15% of affected students believed that supernatural act is responsible for such outbreak but no study was found to reflect the perception of the guardians at the time of outbreak [24]. Prompt public identification of episodes of mass psychogenic illness has been advocated as an important step in terminating them, but such an approach can be problematic in practice [9, 20]. Prompt recognition, coordinated investigations, effective stress coping strategy, and environmental modifications are essential in alleviation of the widespread anxiety surrounding an episode of mass psychogenic illness [19]. Awareness of the characteristics of mass psychogenic illness is crucial for physicians and other healthcare personnel who respond to such outbreaks [1].

## 6. Conclusion

Mostly adolescents are endangered in mass psychogenic illness with typically medically unexplained somatic complains creating an emergency. In dealing with a mass psychogenic illness, a prompt, coordinated response is important in resolving widespread community anxiety surrounding these episodes.

## Figures and Tables

**Table 1:** Age distribution (*n* = 93).

Age in years	Number of students	Percentage
11	9	10%
12	14	15%
13	32	34%
14	26	28%
≥15	9	13%

**Table 2:** Exposure to probable trigger factor among students.

Probable Trigger Factor	Percentage of Students
Consumption of cake	98%
Noticed abnormal smell or taste or both in the cake	88%
Came to school without taking breakfast	84%
Felt ill while handling ill students to transfer to hospital	63%
Felt ill by only seeing other felt ill in the school	20%
Became ill while watching television telecast of the incidence at home	11%
Felt ill spontaneously while playing	05%

**Table 3:** Symptoms profile of ill students (*n* = 93).

Symptoms	No of students	Percentage
Abdominal pain	77	82.8%
Headache	68	73.1%
Body ache	59	63.4%
Nausea	64	68.8%
Chest pain	64	68.8%
Generalized weakness & fatigue	57	61.3%
Burning body	54	58.1%
Dizziness	42	45.2%
Dry mouth	29	31.2%
Hyperventilation	23	24.7%
Breathlessness	18	19.4%
Throat burning	15	16.1%
Crying & shouting	15	16.1%
Muscle cramp	13	14.0%
Salivation	10	10.8%
Vomiting	8	8.6%
Pseudo seizure	7	7.5%
Cold extremity	6	6.5%
Sweating	5	5.4%
Unconsciousness	4	4.3%
Limb weakness	3	3.2%
Visual disturbance	2	2.2%
